# FAM-related prognostic molecular subtype screening identified epithelial-derived *MAOA*-inhibiting bladder cancer

**DOI:** 10.3389/fcell.2026.1732999

**Published:** 2026-02-27

**Authors:** Hui Yu, Qingqiang Lei, Wenyong Yang, Min Cao, Miaoyu Zhang, Taisong Wang, Junhao Dong, Xuerui Chen, Xu Su, Yi Huang, He Xu, Hui Zhuo, Liangbin Lin

**Affiliations:** 1 Department of Urology, The Affiliated Hospital of Southwest Jiaotong University, The Third People’s Hospital of Chengdu, Chengdu, China; 2 Center of Bone Metabolism and Repair, Department of Wound Repair and Rehabilitation Medicine, State Key Laboratory of Trauma, Burns and Combined Injury, Trauma Center, Research Institute of Surgery, Daping Hospital, Army Medical University, Chongqing, China; 3 Department of Neurosurgery, The Affiliated Hospital of Southwest Jiaotong University, The Third People’s Hospital of Chengdu, Chengdu, China; 4 Medical Research Center, The Affiliated Hospital of Southwest Jiaotong University, The Third People’s Hospital of Chengdu, Chengdu, China; 5 Obesity and Metabolism Medicine-Engineering Integration Laboratory, Department of General Surgery, The Affiliated Hospital of Southwest Jiaotong University, The Third People’s Hospital of Chengdu, Chengdu, China; 6 The Center of Gastrointestinal and Minimally Invasive Surgery, Department of General Surgery, The Affiliated Hospital of Southwest Jiaotong University, The Third People’s Hospital of Chengdu, Chengdu, China

**Keywords:** bladder cancer, fatty acid metabolism, *MAOA*, prognosis prediction, single-cell RNA-seq

## Abstract

**Introduction:**

Fatty acid metabolism (FAM) is essential for cancer cell proliferation and progression, contributing to membrane synthesis, energy storage, and signaling molecule production. However, effective therapeutic strategies targeting FAM are yet to be established in clinical practice. This study aimed to develop a novel FAM-related prognostic signature for bladder cancer (BLCA) and investigate its biological and clinical significance.

**Methods:**

We analyzed 359 BLCA samples and constructed a four-gene FAM-RiskScore (FAMR) signature based on FAM-related genes. Unsupervised clustering was performed to classify BLCA into molecular subtypes. The FAMR model was validated using internal and external cohorts. Functional enrichment, immune infiltration, and single-cell RNA sequencing analyses were conducted to explore underlying biological mechanisms. In vitro experiments, including proliferation and migration assays, were performed in T24 and 5637 bladder cancer cells following MAOA knockdown.

**Results:**

BLCA samples were classified into two subtypes (C1 and C2), with C1 showing better overall survival, enhanced steroid metabolism, downregulated chemokine signaling, and lower immune scores. The FAMR signature comprising PATZ1, TTC6, AEBP1, and MAOA was established. High FAMR scores–associated with low PATZ1, TTC6, MAOA, and high AEBP1 expression–predicted poor prognosis. FAMR positively correlated with pathways related to chemotaxis, inflammation, and cytoskeleton regulation, but negatively with fatty acid metabolism pathways. Higher FAMR scores were observed in females, patients aged >60, and advanced-stage tumors. Single-cell analysis revealed AEBP1 was mainly expressed in cancer-associated fibroblasts, while MAOA was enriched in cancer cells. Functional studies demonstrated that MAOA knockdown significantly enhanced proliferation and migration of bladder cancer cells in vitro.

**Discussion:**

We developed and validated a novel FAM-related risk signature that effectively predicts prognosis in BLCA. Our findings highlight MAOA as a potential tumor suppressor in bladder cancer, warranting further investigation as a therapeutic target. This FAMR model may facilitate risk stratification and inform personalized treatment strategies for bladder cancer patients.

## Introduction

Bladder cancer (BLCA) is among the ten most prevalent cancers worldwide, with considerable morbidity and mortality, imposing a substantial burden on healthcare systems (Röhrig and Schulze, 2016). BLCAs can be categorized into two major groups: muscle-invasive bladder cancers (MIBCs) and non-muscle-invasive bladder cancers (NMIBCs). MIBCs represent those that have spread into or through the detrusor muscle and account for approximately 25% of the newly diagnosed BLCA patients, whereas NMIBCs are restricted to the mucosa or submucosal connective tissue and account for approximately 75% of the newly diagnosed BLCA patients (Tran et al., 2021). Patients with MIBC have a poor prognosis, with a 5-year overall survival of 49%. Patients with NMIBC have a good life expectancy, with a 5-year BLCA-specific mortality of 0.5%, 1.7%, and 6.8% among grade 1, 2, and 3 tumors, respectively (Lenis et al., 2025). However, patients with NMIBC experience a high rate of disease recurrence within 1 and 5 years post-transurethral resection of bladder tumor (TURBT) (15%–61% and 31%–78%, respectively), along with progression to MIBC (10%–40% of high-risk NMIBCs) (Migita et al., 2008). The frequent recurrence of NMIBC results in lifelong cystoscopic surveillance and multiple therapeutic interventions, placing a heavy burden on public health systems. Therefore, the prognostic molecular features need to be elucidated for optimizing clinical treatment.

Cancer cells often exhibit metabolic perturbations that support cell growth and proliferation, which require fatty acids (FAs) for the synthesis of membranes, energy storage, and the production of signaling molecules. FAs consist of a terminal carboxyl group and a hydrocarbon chain that have different lengths and degrees of desaturation. The synthesis of FAs is the process that converts nutrients into metabolic intermediates (Röhrig and Schulze, 2016). Diverse studies indicted the crucial role of FA metabolism (FAM) in cancer cell proliferation. For example, ATP citrate lyase (ACLY) converts citrate into oxaloacetate and two-carbon acetyl-CoA, which is the precursor for FA synthesis. *ACLY* knockdown prevents xenograft tumor formation by human cancer cells (Migita et al., 2008; Bauer et al., 2005). Acetyl-CoA carboxylase 1 (ACC1) carboxylates acetyl-CoA to form malonyl-CoA, thus providing a substrate for FA synthesis (Currie et al., 2013). The knockdown of *ACC1* induces apoptosis of prostate cancer cells (Brusselmans et al., 2005). Given the critical role of FAM in cancer proliferation and progression, targeting FAM might be a therapeutic strategy. For example, the inhibition of sterol regulatory element-binding protein 1 (SREBP-1), the master transcriptional regulators of FA synthesis, causes marked reduction of cellular growth in cancer cells (Williams et al., 2013). Inhibiting acyl-CoA synthetases, which are enzyme families that are responsible for the activation of intracellular free FAs, reduces the production of cardiolipins, thus leading to apoptosis of cancer cells (Mashima et al., 2005). Using the synthetic compound C75 to inhibit the β-ketoacyl-reductase activity of fatty acid synthase (FASN) triggers apoptosis in several cancer cell lines (Li et al., 2001; Zhou et al., 2003; Menendez et al., 2005) and shows anti-tumorigenic effects in mesothelioma (Gabrielson et al., 2001), breast (Pizer et al., 2000), renal (Horiguchi et al., 2008), lung (Relat et al., 2012), and prostate cancer (Chen et al., 2012) xenograft models.

FAM has received substantial attention in cancer therapy, but strategies targeting this process have not yet translated into clinical practice. In this study, we identified the expression and significance of FAM-characteristic genes in BLCA. Based on the FAM-related gene set, the two molecular subtypes of BLCA were clustered. We next analyzed the prognosis, clinical features, and biological functions of the two clusters. Through univariate Cox regression analysis of differentially expressed genes (DEGs), we identified a four-gene signature (*PATZ1*, *TTC6*, *AEBP1*, and *MAOA*) model for the prognostic prediction of BLCA called FAM-RiskScore (FAMR). The high value of FAMR was correlated with poor prognosis, along with low expression of *PATZ1*, *TTC6*, and *MAOA*, and high expression of *AEBP1.* We validated the FAMR model using an external validation cohort and analyzed the correlation between FAMR and biological functions as well as clinical features. Single-cell RNA-seq data revealed distinct cellular origins of *AEBP1* and *MAOA*, with *AEBP1* predominantly expressed in cancer-associated fibroblasts (CAFs) and *MAOA* primarily derived from cancer cells. Functional assays demonstrated that *MAOA* knockdown significantly enhanced the proliferative and migratory capacities of human BLCA cells, as evidenced by *in vitro* experiments using the 5637 and T24 cell lines. Collectively, in this study, we establish a novel FAMR model based on FAM-related genes for prognostic prediction of BLCA, providing a potential framework for the development of targeted therapeutic strategies in clinical settings.

## Materials and methods

### Data acquisition and preprocessing

The tissue expression data and clinical information of BLCA patients were downloaded from The Cancer Genome Atlas (TCGA) database (https://www.cancer.gov/ccg/research/genome-sequencing/tcga) and the ArrayExpress database (https://www.ebi.ac.uk/biostudies/arrayexpress). The dataset underwent the following processing steps: (1) exclusion of samples lacking clinical follow-up information; (2) conversion of gene identifiers to gene symbols; (3) selection of the highest expression value in cases of multiple entries for the same gene symbol; (4) removal of samples with missing expression profile data. After preprocessing, 359 samples were obtained from TCGA-BLCA and 476 samples were obtained from the ArrayExpress database (Table 1).

**TABLE 1:** Information of the cohorts.

Clinical features	​	TCGA-BLCA	Array express
OS	0	197	367
	1	162	109
DSS	0	237	​
	1	110	​
Grade	Low grade	14	​
	High grade	342	​
Stage	Stage I	2	​
	Stage II	113	​
	Stage III	124	​
	Stage IV	118	​
Gender	Male	267	​
	Female	92	​
Age (years)	>60	264	​
	≤60	95	​

We retrieved three *Homo sapiens*-derived BLCA tissues with comprehensive staging information from the GEO database (GSE129845) and analyzed their single-cell RNA sequencing data using the R package Seurat. Quality control filters excluded cells with fewer than 200 genes or over 15% mitochondrial genes, resulting in the retention of 13,490 cells for further analysis. Data normalization was achieved using the LogNormalize method, and batch effects were mitigated with the Harmony package. The FindVariableFeatures function identified the top 2,000 variable genes. Dimensionality reduction was performed through principal component analysis, t-SNE, and UMAP, followed by cell clustering using the FindNeighbors and FindClusters functions. Cell annotation was facilitated by R package SingleR.

Genes related to the FAM pathways (HALLMARK_FATTY_ACID_METABOLISM) were obtained from the Molecular Signature Database v7.0 (MSigDB). A total of 158 FAM-related genes were included in the analysis (Supplementary Table S1).

### Consistent cluster analysis

The TCGA expression profile data underwent filtration to exclude genes with expression levels below one in more than 50% of samples, followed by univariate Cox analysis to identify prognostic-related FAM genes using a significance threshold of p < 0.05. ConsensusClusterPlus (v1.48) was utilized for uniform clustering of TCGA samples, utilizing D2 and Euclidean distance as the clustering algorithm and measure, with parameters set as pFeature = 1, rep = 100, distance = “Spearman,” and pItem = 0.8. The limma package was used to assess molecular subtype discrepancies and conduct functional enrichment analysis, whereas DAVID was implemented to assess significantly enriched pathways, including KEGG and GO pathways, across distinct BLCA groups. Pathways were considered enriched if they met the criteria of p < 0.05 and a false discovery rate (FDR) < 0.05.

### Immune score calculation

To analyze tumor immune infiltration, we performed a multi-algorithm assessment using the TCGA-BLCA cohort normalized expression matrix [log2(TPM+1)]. First, stromal and immune scores were globally evaluated via the ESTIMATE package (v1.0.13) under default parameters. Next, immune cell subtype deconvolution was conducted using CIBERSORT with the LM22 signature and 1,000 permutations, retaining only samples with a CIBERSORT output p-value <0.05. Additionally, absolute immune and stromal cell abundances were quantified using the MCP-counter package (v1.2.0) and its predefined signatures. Between-group differences were assessed via the Wilcoxon rank-sum test; pairwise correlations were examined with Spearman’s rank correlation. For all multiple comparisons, the false discovery rate (FDR) was controlled using the Benjamini–Hochberg procedure, with an adjusted p-value (FDR) < 0.05 being considered significant. To enhance robustness, final interpretations focused exclusively on immune infiltration signals that were consistently observed across all three computational methods (ESTIMATE, CIBERSORT, and MCP-counter).

### Development of a prognostic risk model utilizing FAM-related gene signatures

Accurate prognosis is crucial for tailoring cancer treatments and improving patient outcomes. Traditional clinical factors offer limited precision, but molecular signatures can enhance prognostic accuracy by capturing tumor diversity.

Our study developed a predictive risk model using FAM-related genes. We began by extracting FAM gene expression data from a public dataset. Univariate Cox regression identified survival-associated FAM genes, which were further refined through LASSO regression to prevent overfitting. We then constructed a prognostic model using multivariate Cox regression with the selected genes. Patient risk scores were calculated based on gene expression levels and model coefficients. Patients were categorized into high- and low-risk groups using the median risk score.

We evaluated the model’s performance using ROC and Kaplan–Meier analyses. This FAM gene-based risk model aims to improve survival predictions and inform personalized treatment strategies for cancer patients.

### Partitioning of training and test sets

We performed bootstrap resampling (1,000 iterations) on our training cohort to obtain more reliable performance estimates. The results showed a C-index of 0.604 (SD: 0.006), with a 95% confidence interval of 0.591–0.611. In the study, the 359 samples from the TCGA dataset were randomly divided into a training set (180 samples) and a test set (179 samples). Training and test sets were selected based on two criteria: balanced patient demographics and clinical outcomes and similar distribution of binary samples after gene expression clustering.

### LASSO cox regression analysis

LASSO regression was used to identify prognostic genes and optimize the risk model. This method reduces coefficients and selects relevant variables, effectively addressing multicollinearity and promoting sparsity in the data. LASSO Cox regression analysis was performed using the glmnet R-package. The optimal model was selected through five-fold cross-validation, and the number of target genes was determined based on confidence intervals at each lambda value.

### RNA extraction and qPCR

Total RNA was extracted from the T24 and 5637 cells using TRIzol reagent (Invitrogen) and reverse-transcribed into cDNA with the kit of HiScript II Q RT SuperMix for qPCR (Vazyme). The cDNA was subjected to qPCR analysis using the Universal SYBR qPCR Master Mix (Vazyme).

### CCK-8

T24 and 5637 cells were seeded in 96-well plates at a density of 3,000 cells per well in Dulbecco’s Modified Eagle Medium (DMEM) supplemented with 10% fetal bovine serum (FBS) and 1% penicillin–streptomycin, respectively. After 48 and 72 h of incubation, 10 μL of Cell Counting Kit-8 (CCK-8, BioSharp) was added to each well, followed by incubation at 37 °C in the dark for 1 h. Cellular viability was assessed by measuring the absorbance at 450 nm. All experiments were performed in triplicate to ensure reproducibility.

### Wound healing

T24 and 5637 cells were seeded in 6-well plates and cultured until a confluent monolayer was formed. A uniform scratch was introduced into the monolayer using a sterile 200-μL pipette tip. The cells were then washed thrice with PBS to remove detached cells and debris, followed by the addition of DMEM supplemented with 10% FBS and 1% penicillin–streptomycin. Wound healing progression was monitored and photographed at 0, 24, and 48 h using an IX71 inverted microscope (Leica Corporation). The images were analyzed using ImageJ software (v1.80), and the wound closure was calculated using the following formula: [(area at 0 h – area at 24/48 h)/area at 0 h] × 100. All the experiments were performed in triplicate to ensure reproducibility.

### Statistical analysis

For the analysis of two or more continuous variables with normal distribution, t-tests or analysis of variance (ANOVA) were applied. Statistical significance was determined by an adjusted p-value <0.05.

## Results

### Flowchart of this study

**FIGURE 1:**
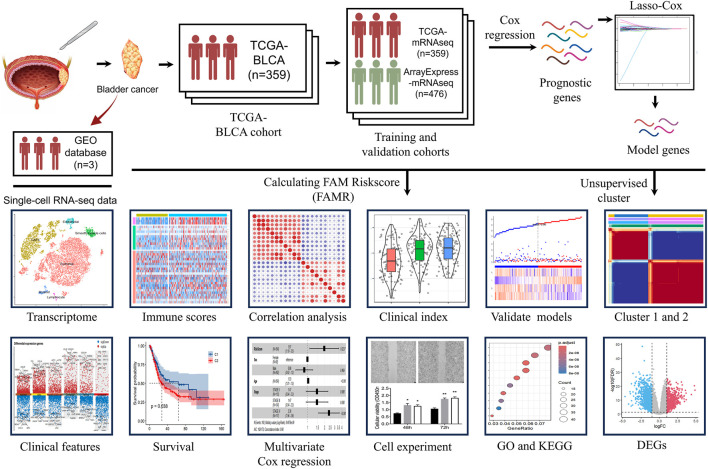
Scheme of the overall strategy of this study.

A total of 359 patients were identified from the TCGA-BLCA database as the training and validation cohort, and 476 patients were identified from the ArrayExpress-mRNAseq-E-MTAB-4321 database as the external validation cohort. A total of 158 FAM-related genes were collected from the Molecular Signature Database v7.0 (MSigDB). The workflow of this study is shown in Figure 1.

### Identification of molecular subtypes based on FAM-related gene and clinical features

**FIGURE 2:**
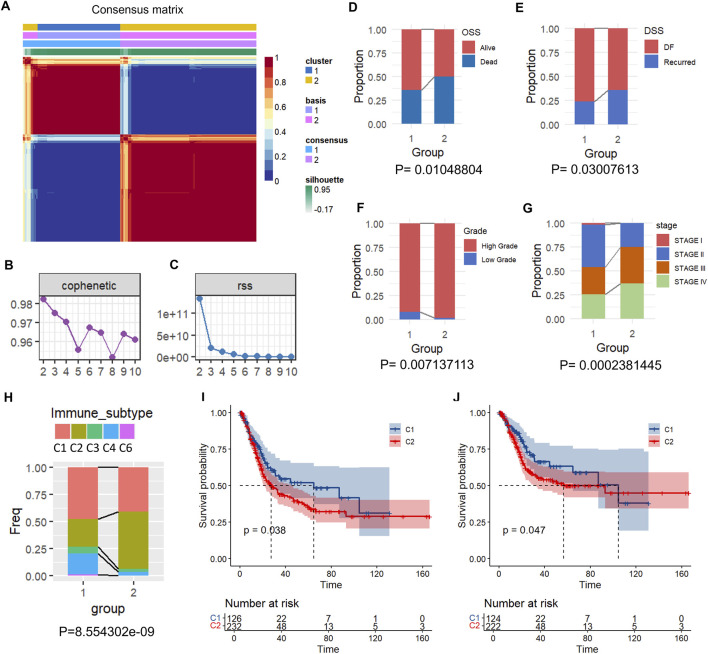
Cluster and prognosis analysis of BLCA based on FAM-related gene. **(A)** Consensus matrix plot of NMF clustering. **(B)** Cophenetic distribution of rank = 2–10, which reflects the stability of the cluster in A. **(C)** RSS distribution at rank = 2–10, of which a higher value indicates more stable clusters. **(D–H)** Distribution of C1 and C2 in the indicated clinical features and immune subtypes. **(I,J)** KM curves of OS **(I)** and DSS **(J)** of the C1 and C2 clusters.

We first obtained the expression of 158 FAM-related genes from the TCGA-BLCA expression profile data. A total of 18 genes associated with BLCA prognosis (p < 0.05) were identified through univariate Cox analysis by R (Supplementary Table S2). Based on the expression of the 18 genes, we clustered BLCA patients using non-negative matrix factorization (NMF). We chose the optimal clustering of k = 2 by synthesizing and the residuals sum of squares (RSS), and two clusters (C1 and C2) were obtained (Figures 2A–C). We then compared the clinical characteristics of the two clusters and found that C1 had a higher survival rate than C2 (Figures 2D,E). In addition, high-grade tumor was more enriched in C2 than in C1, indicating an enhanced cancer progression (Figure 2F). C1 had more early-stage tumors, including stages I and II, and less late-stage tumors, including stages III and IV, than C2, all of which indicated a better prognosis of C1 (Figure 2G).

Thorsson et al. (2018) identified six types of inter-tumoral immune stages: cluster 1 (wound healing), cluster 2 (IFN-γ dominant), cluster 3 (inflammatory), cluster 4 (lymphocyte depleted), cluster 5 (immunologically quiet), and cluster 6 (TGF-β dominant), of which cluster 3 has the best prognosis. We found that C1 was more correlated with clusters 1, 3, 4, and 5, whereas C2 was more correlated with cluster 2 (Figure 2H), indicating a better prognosis of C1, which is consist with the Kaplan–Meier (KM) curves. C1 showed a better prognosis indicated by overall survival (OS) and disease-specific survival (DSS) (Figures 2I,J).

### C1 has a lower immune score

**FIGURE 3:**
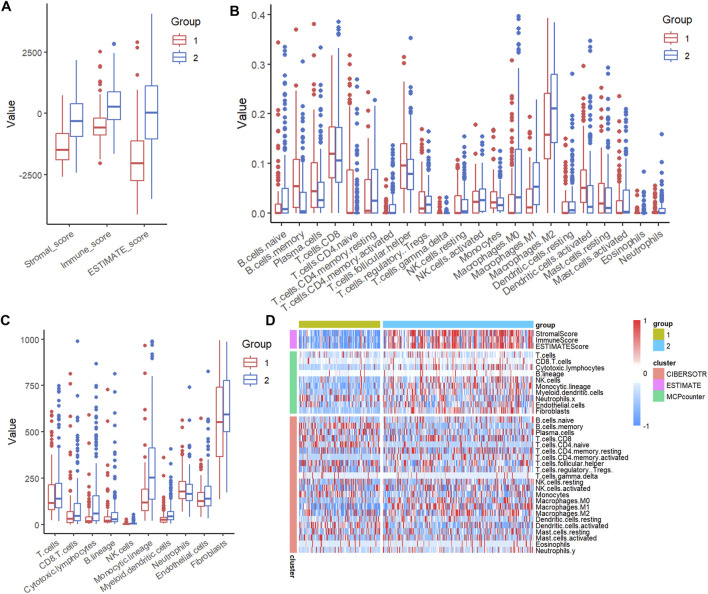
C1 has lower immune score. **(A)** ESTIMATE analysis of immune scores of C1 and C2. **(B)** CIBERSORT analysis of immune scores of CA and C2. **(C)** MCPcounter analysis of immune scores of C1 and C2. **(D)** Heatmap of the indicated immune scores of C1 and C2.

We next evaluated the immune scores of C1 and C2 using the ESTIMATE package in R software. The result showed that C1 had a lower immune score, stromal score, and ESTIMATE score than C2 (Figure 3A). Further studies using R software packages CIBERSORT and MCPcounter indicated that C1 had higher scores for memory B cells, plasma cells, follicular helper T cells, and activated dendritic cells, whereas it had lower scores for M0 macrophage, M2 macrophages, monocytic lineage, and cytotoxic lymphocytes (Figures 3B,C). Heatmap also showed the lower immune score of C1 and different specific immune cell scores between C1 and C2 (Figure 3D).

### C1 is correlated with enhanced steroid metabolism and downregulated chemokine signaling

**FIGURE 4:**
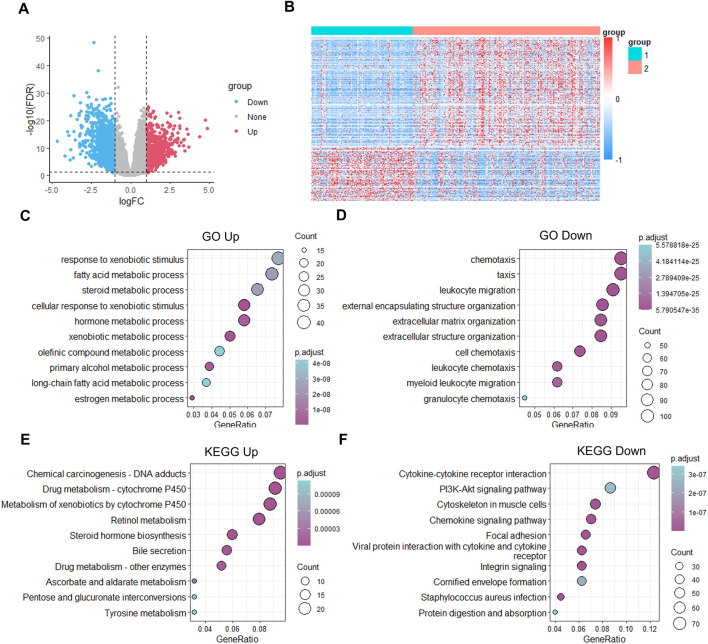
Biological functional analysis of the C1 and C2 subtypes. **(A)** Volcano map of the differently expressed genes in C1. **(B)** Heatmap of the differently expressed genes in C1 and C2. **(C,D)** Biological processes of the differently upregulated **(C)** and downregulated **(D)** genes in C1. **(E,F)** KEGG annotation of the differently upregulated **(E)** and downregulated **(F)** genes in C1.

To verify the biological characteristics between the two clusters, we next calculated the DEGs between C1 and C2 using the limma package and applied a filter with the criteria of |log2FC| > 1 and FDR < 0.01. The results showed 1,886 DEGs, with 632 upregulated and 1,254 downregulated genes based on C1 (Figures 4A,B). The full list of DEGs is provided in Supplementary Table S3. Pathways associated with steroid metabolic process, response to xenobiotic stimulus, and hormone metabolic process were enriched in C1, whereas pathways related to chemotaxis, leukocyte migration, and extracellular structure organization were downregulated in C1 based on GO functional pathway enrichment analysis (Figures 4C,D). KEGG enrichment analysis showed that pathways associated with drug metabolism, retinol metabolism, and steroid hormone biosynthesis were upregulated in C1, whereas cytokine signaling, PI3K-AKT signaling, and cytoskeleton were downregulated in C1 (Figures 4E,F).

### Construction of a prognostic risk model

**FIGURE 5:**
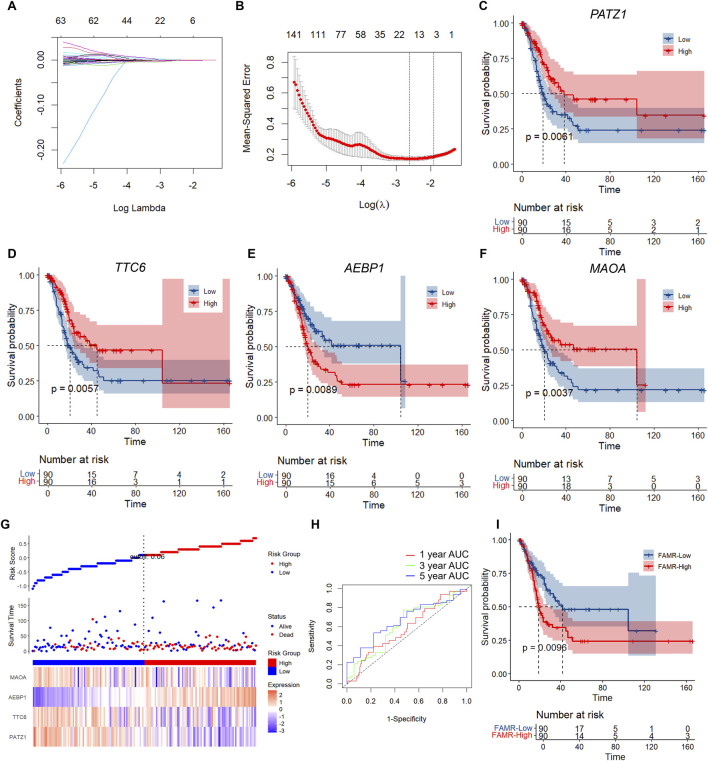
Construction of the four-gene FAM-RiskScore (FAMR) signature. **(A)** LASSO Cox regression analysis of the 295 prognosis-associated genes. The horizontal axis represents the log value of a dependent variable, and the vertical axis represents the coefficient of the independent variable. **(B)** Cross-validation analysis of the confidence intervals under each lambda. **(C–F)** KM curves based on the expression of the indicated genes. **(G)** RiskScore, survival status, and four-gene expression of the TCGA training set. **(H)** ROC curves and AUCs of the four gene features in the TCGA training set. **(I)** KM curves based on the RiskScore in the TCGA training set.

To construct the prognostic risk model, we categorized the 359 patients into training and validation sets randomly, with the training group containing 180 patients and the validation group containing 179 patients. Based on C1 and C2, we identified 1,886 DEGs (Figure 4; Supplementary Table S3). We then identified 295 prognosis-associated genes through univariate regression Cox risk model analysis based on the survival data (with p < 0.01 as the threshold) (Supplementary Table S4). We next applied the R-package glmnet for LASSO Cox regression analysis of the 295 prognosis-associated genes and revealed that the number of coefficients of independent variables gradually increased as lambda increased (Figure 5A). Cross-validation was performed to calculate the confidence intervals under each lambda, and it revealed the optimal model at lambda = 0.07290695, with 16 genes identified (Figure 5B; Supplementary Table S5).

To identify the optimal predictive gene signature, we performed stepwise model selection based on the Akaike information criterion (AIC) using the step AIC function from the MASS package. This procedure began with a full model containing all 16 candidate genes. At each step, the variable whose removal most significantly lowered the AIC value was eliminated, thereby refining the model toward a parsimonious structure without substantially compromising the goodness-of-fit. Through this iterative process, the initial 16-gene set was reduced to a final signature comprising four genes: *PATZ1*, *TTC6*, *AEBP1*, and *MAOA*. *PATZ1*, *TTC6*, and *MAOA* were positively associated with BLCA prognosis, as higher expression of these genes was correlated with better survival outcomes (Figures 5C,D,F). *AEBP1* was negatively associated with BLCA prognosis, as higher expression of the gene had poor survival outcomes (Figure 5E).

We next calculated the RiskScore of the 180 samples in the training cohort based on the expression of the four indicated genes using the ggRISK package. The final four-gene signature formula is as follows: FAMR = [−0.606 × log2(PATZ1+1)] + [0.0594 × log2(TTC6+1)] + [0.5085 × log2(AEBP1+1)] + [−0.0352 × log2(MAOA+1)]. The results showed that higher RiskScore represented poor prognosis (Figure 5G). Higher FAMR was associated with lower expression of *PATZ1*, *TTC6*, and *MAOA* and higher expression of *AEBP1*, which was consistent with the KM curve results (Figure 5G). Then, we evaluated the area under the curve (AUC) of prognostic prediction efficiency at 1, 3, and 5 years by R-wrapper time ROC analysis. The results showed that the AUC values of the indicated models were higher than 0.65 (Figure 5H). Furthermore, the KM analysis showed that the low FAMR group had a better prognosis than the high FAMR group (Figure 5I). These results indicated the clinical application value of FAMR.

### Validation of the risk model

**FIGURE 6:**
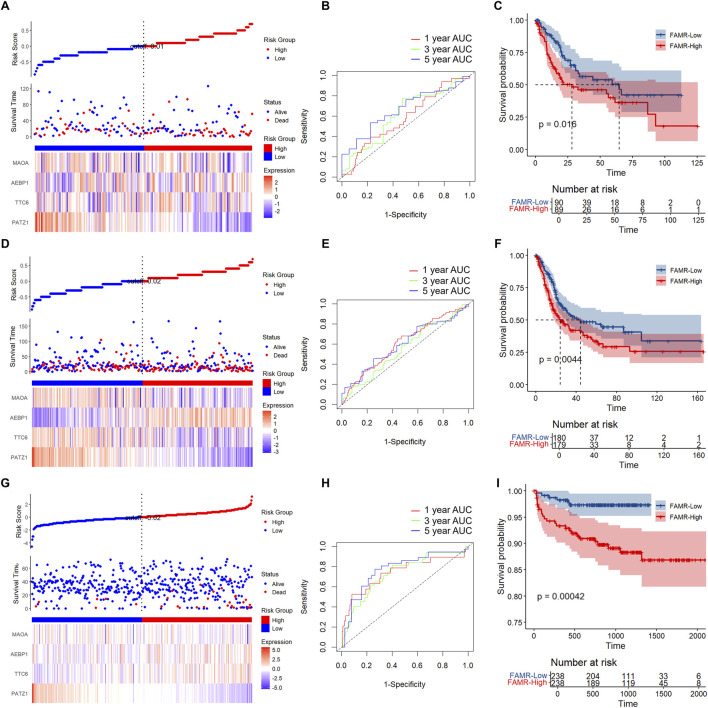
Validation of the four-gene FAM-RiskScore (FAMR) model. **(A,D,G)** RiskScore, survival status, and four-gene expression of the TCGA test set **(A)**, the full TCGA dataset **(D)**, and the external validation cohort from the ArrayExpress-mRNAseq-E-MTAB-4321 database **(G)**. **(B,E,H)** ROC curves and AUCs of the four gene features in the TCGA test set **(B)**, the full TCGA dataset **(E)**, and the external validation cohort **(H)**. **(C,F,I)** KM curves based on the RiskScore in the TCGA test set **(C)**, the full TCGA dataset **(F)**, and the external validation cohort **(I)**.

To verify the FAMR model, we performed analysis using the 179 test patients’ data alone and the data of all 359 patients as the test cohort. Consistently, higher FAMR was correlated with poor prognosis, with lower expression of *PATZ1*, *TTC6*, and *MAOA* and higher expression of *AEBP1* (Figures 6A,D). The AUC of prognostic prediction efficiency at 1, 3, and 5 years was higher than 0.65 in both the 179 and 359 validation cohorts (Figures 6B,E). The KM analysis results also showed that the low FAMR group was associated with a better prognosis compared with the high FAMR group (Figures 6C,F). To further validate the FAMR model using the external validation cohort, we collected data of the 476 patients from the ArrayExpress-mRNAseq-E-MTAB-4321 database and performed the FAMR analysis. The results showed that higher FAMR was associated with poor prognosis based on the external validation cohort (Figure 6G). The AUC of prognostic prediction efficiency at 1, 3, and 5 years was higher than 0.75 (Figure 6H). Consistently, the low FAMR group had a significantly better prognosis than the high FAMR group, as shown by the KM analysis (Figure 6I). Together, we verified the FAMR model using internal and external validation cohorts, and the results showed the stability and universality of the model.

### Correlation analysis of FAMR with biological functions and clinical features

**FIGURE 7:**
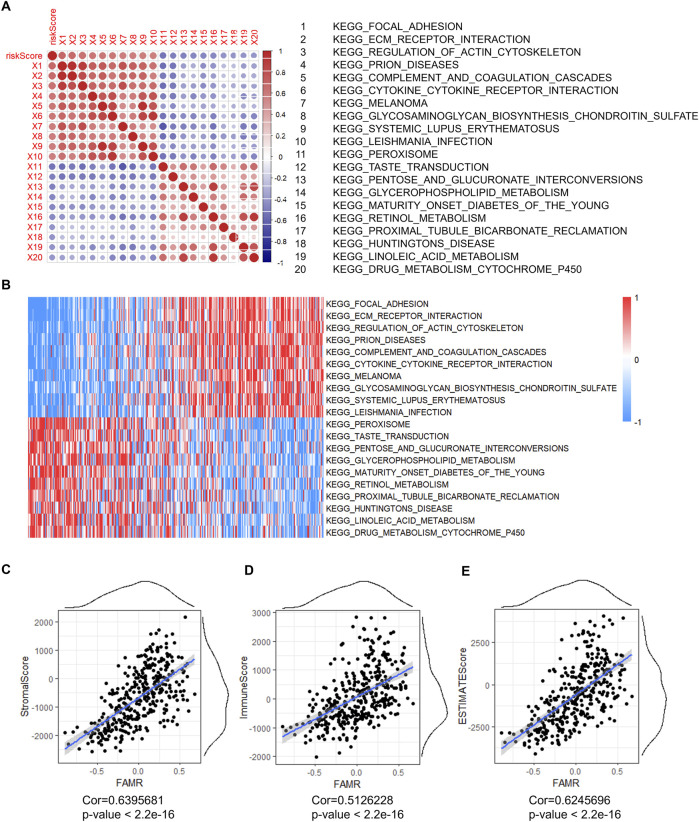
Correlation analysis of FAMR with biological functions. **(A)** Top 10 significantly positively and negatively correlated pathways with FAMR. **(B)** Heatmap of the top 10 significantly positively and negatively correlated pathways. **(C–E)** Correlation between FAMR and StromalScore **(C)**, ImmuneScore **(D)**, and ESTIMATEScore **(E)**.

We next studied the corresponding gene expression profiles of the GSEA to analyze the correlation between FAMR and biological functions. The ssGSEA scores of each sample were calculated, and the correlation between the FAMR and biological function was further analyzed. The results showed the top 10 significantly positively and negatively correlated pathways (Figures 7A,B). The positively correlated pathways included KEGG_FOCAL_ADHERION, KEGG_ECM_RECEPTOR_INTERACTION, KEGG_REGULATION_OF_ACTIN_CYTOSKELETON, and KEGG_PRION_DISEASES, among others, which were associated with chemotaxis, cytoskeleton, infections, and inflammations (Figures 7A,B). The negatively correlated pathways included KEGG_PEROXISOME, KEGG_TASTE_TRANSDUCTION, KEGG_GLYCEROPHOSPHOLIPID_METABOLISM, KEGG_RETINOL_METABOLISM, and KEGG_LINOLEIC_ACID_METABOILISM, among others, which were associated with fatty acid metabolism and metabolism-related diseases (Figures 7A,B). Furthermore, we assessed the correlations between FAMR and ImmuneScore, and the results revealed that the ImmuneScore, StromalScore, and ESTIMATEScore were positively related with FAMR (Figures 7C–E).

**FIGURE 8:**
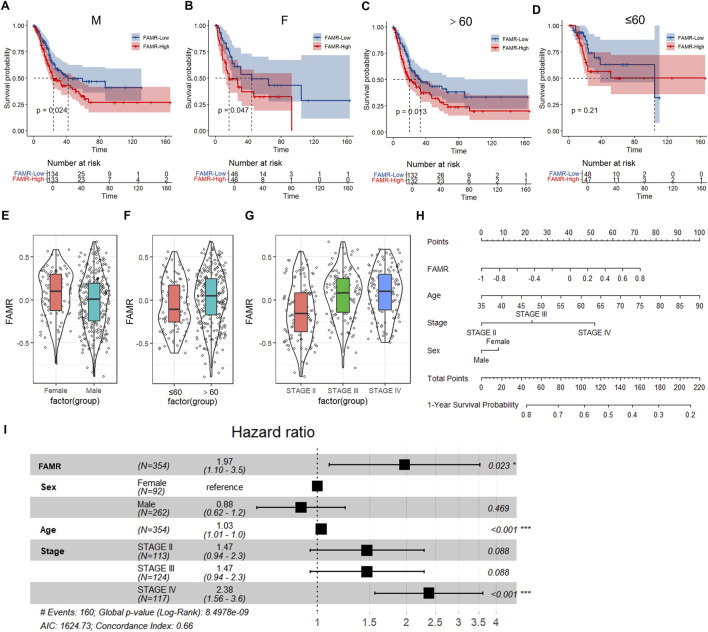
Correlation analysis of FAMR with clinical features. **(A–D)** FAMR-based KM curves of male patients **(A)**, female patients **(B)**, >60 patients **(C)**, and ≤60 patients **(D)**. **(E–G)** Value of FAMR based on groups of gender **(E)**, age **(F)**, and tumor stage **(G)**. **(H)** Nomogram provides a method to calculate OS from the FAMR. To use it, we locate the ‘FAMR’ axis and draw a line straight up to the “points” axis to determine the score associated to the regimen. The process is repeated for the three other variables: sex, age, and stage. The scores are added, and the total score is located on the “total points” axis. **(I)** Forest plot of multiple Cox regression analysis.

Additionally, we investigated whether gender, age, and clinical features such as the cancer stage affected FAMR. Therefore, KM analyses were performed in male female patients, as well as in patients aged >60 years and ≤60 years. The results showed that the low FAMR groups all had better prognosis than the high FAMR groups in the four indicated groups (Figures 8A–D), indicating the stable indicative ability of the FAMR model. We next analyzed the FAMR in the gender, age, and tumor stage groups and found that the female group had higher FAMR (Figure 8E), indicating a poor prognosis for females, which was consistent with the information that BLCA in women is often diagnosed at a higher stage with worse prognosis (Dobruch et al., 2025). The >60 group had higher FAMR (Figure 8F), which was consistent with the information that the elderly are more susceptible to BLCA (Van Hoogstraten et al., 2023). Furthermore, FAMR increased from stages II to IV, indicating worse prognosis along with cancer progress (Figure 8G). We further revealed that FAMR was correlated with the survival rate of BLCA by multivariate Cox regression analysis (Figures 8H,I). In addition to FAMR, factors such as the patient age, gender, and disease stage also exert varying degrees of influence on the prognosis of BLCA. Integrating these clinical variables with FAMR may thus serve as a more robust approach for predicting outcomes in BLCA patients.

### Single-cell RNA-seq data identified that *AEBP1* is expressed by CAFs and *MAOA* by epithelial cells in the tumor microenvironment

**FIGURE 9:**
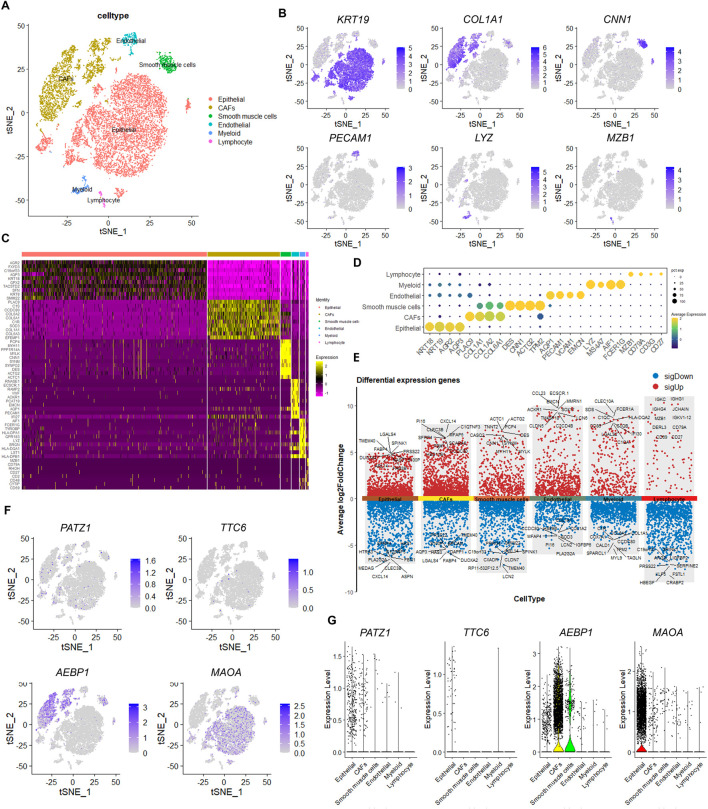
Single-cell RNA-seq data analyses of the expression of *AEBP1* and *MAOA*. **(A)** t-Distributed stochastic neighbor embedding (t-SNE) plot visualization of cell clusters in the three patients with bladder cancer colored by the cell types. **(B)** Expression levels of the indicated genes. **(C)** Heatmap of differentially expressed genes (DEGs) of the indicated cell types. **(D)** Dot plot showing the expression level of DEGs of all the cell types. **(E)** Volcano plot illustrating the DEGs among the six cell types. **(F)** Expression of the indicated genes among the six cell types colored by the expression levels. **(G)** Violin plots representing the expression of the indicated genes of the six cell types.

**FIGURE 10:**
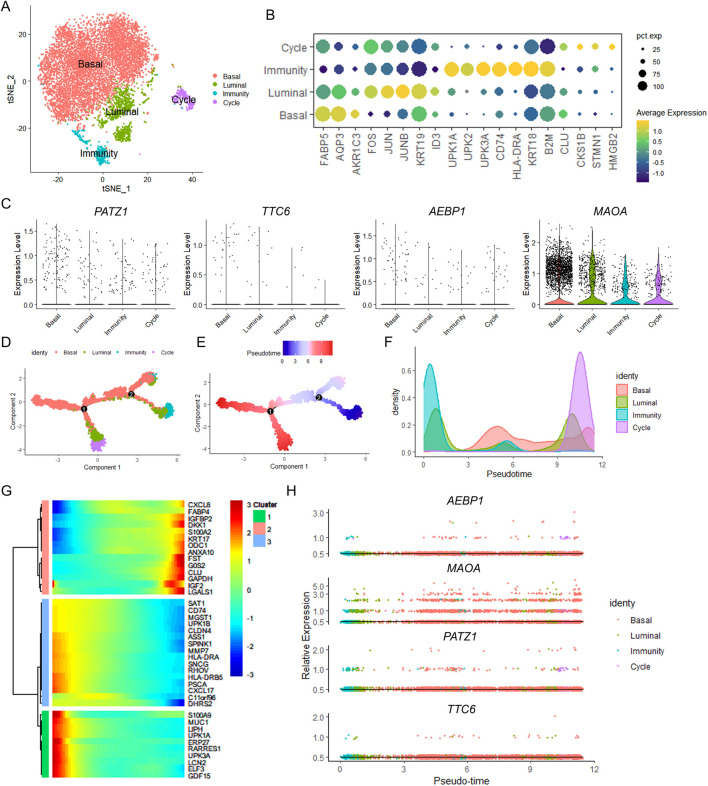
Pseudo-time analysis of the epithelial subpopulations. **(A)** t-SNE plot showing re-clustering of epithelial cells. **(B)** Dot plot showing the expression level of DEGs of the epithelial subpopulations. **(C)** Violin plots representing the expression of the indicated genes of the epithelial subpopulations. **(D–F)** Pseudo-time analysis of epithelial subpopulations inferred by Monocle 2. The epithelial subpopulations **(D)**, pseudo‐temporal ordering **(E)**, and cell density plot **(F)** were labeled by colors. **(G)** Heatmap showing the top 40 genes expressed with the pseudo-time trajectory of epithelial subpopulations. **(H)** Trajectory of the expression of *PATZ1*, *TTC6*, *AEBP1*, and *MAOA*.

The tumor microenvironment (TME) consists of diverse cell types, including cancer cells, stromal, and immune cells, which interact with each other and play critical roles in cancer. To deconvolute the cellular source of the four indicated FAMR-related genes, we analyzed their expression in publicly available single-cell RNA-seq datasets from three patients with BLCA. A total of six cell types were identified, encompassing epithelial cells, cancer-associated fibroblasts (CAFs), smooth muscle cells (*CNN1*, *DES*), endothelial cells (*PECAM1*, *AQP1*), myeloid cells (*LYZ*, *MS4A7*), and lymphocytes (Figures 9A–D). Epithelial cells were characterized by the high expression of *KRT19*, *AGR2*, and *AQP3* (Figures 9C–E). CAFs were characterized by the high expression of *COL1A1*, *PLAC9*, *C1R*, and *CXCL14* (Figures 9C–E). *MZB1*, *CD79A*, and *CD3G* were enriched in lymphocytes, indicating that this population encompassed B cells and T cells (Figures 9C–E). We next analyzed the expression of the four indicated FAMR-related genes and found that *AEBP1* was mainly expressed by CAFs and some of the smooth muscle cells (Figures 9F,G). The expression of *MAOA* was mainly contributed by epithelial cells, indicating their cancer cell-derived origin (Figures 9F,G). In addition, *PATZ1* and *TTC6* were rarely expressed in those three patients within the BLCA dataset (Figures 9F,G). These data indicated that the prognosis-related genes *AEBP1* and *MAOA* were CAF-derived and cancer cell-derived, respectively. Furthermore, we categorized 8,258 BLCA epithelial cells into four clusters based on the DEGs among different BLCA epithelial cell sub-clusters. A total of four cell types were identified, encompassing basal (*FABP5* and *AQP3*), luminal (*JUN* and *JUNB*), immunity (*UPK1A* and *UPK2*), and cycle (*STMN1* and *HMGB2*) (Figures 10A,B). *MAOA* is highly expressed in epithelial cells and is present across all four epithelial cell subpopulations (Figure 10C). Pseudo‐time analysis revealed that the four sub-clusters were distributed along an evolutionary pathway: immunity→ luminal/cycle/basal (Figures 10D–G). The expression of genes such as *PATZ1*, *TTC6*, *AEBP1*, and *MAOA* does not change with the pseudo-time progression of epithelial cells (Figure 10H).

### *MAOA* knockdown enhances the proliferation and migration of T24 and 5637 bladder cancer cells

**FIGURE 11:**
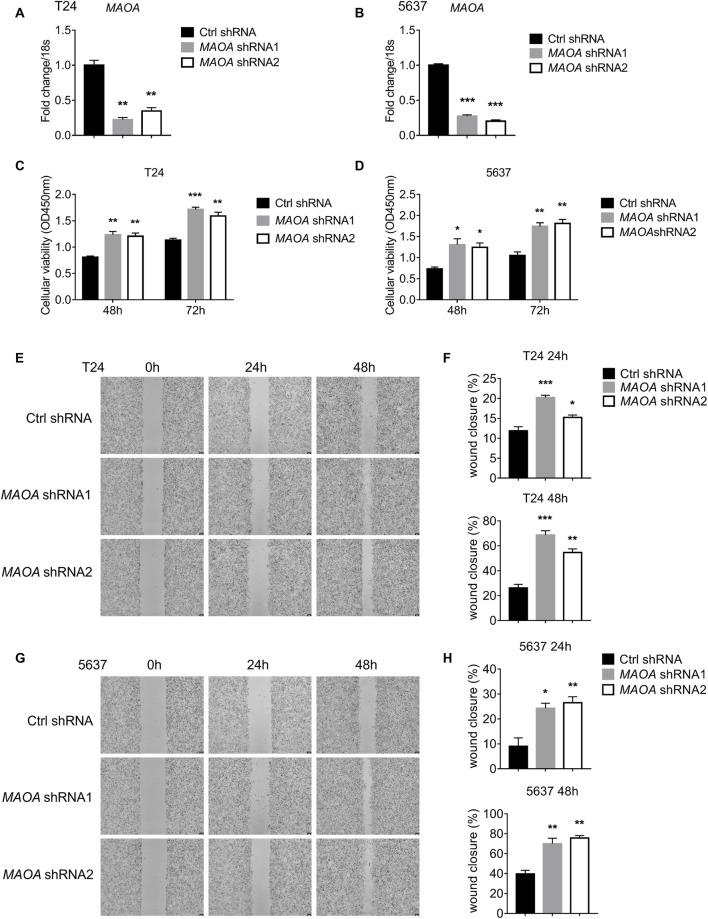
*MAOA* knockdown enhances the proliferation and migration of T24 and 5637 bladder cancer cells. **(A,B)** qPCR assays to detect the expression of *MAOA* in T24 **(A)** and 5637 **(B)** cells. **(C,D)** CCK-8 assays to monitor the proliferation of T24 **(C)** and 5637 **(D)** cells at 48 and 72 h **(E–H)** Wound healing assays to detect the migration abilities of T24 **(E)** and 5637 **(G)** cells at 0, 24, and 48 h, and the summary of the wound closure of the indicated cells **(F,H)**.

To validate the function of the four identified genes in BLCA prognosis, we chose *MAOA*, the only gene predominantly expressed by epithelial tumor cells, for *in vitro* functional validation. We first silenced the expression of *MAOA* in human BLCA cell lines T24 and 5637 using short hairpin RNA (shRNA) (Figures 11A,B). Cell Counting Kit-8 (CCK-8) assays showed that knockdown of *MAOA* significantly promoted the proliferation of both T24 and 5637 cells (Figures 11C,D), indicating the inhibitory role of *MAOA* in BLCA proliferation. In addition, *MAOA* knockdown also resulted in enhanced migration of T24 (Figures 11E,F) and 5637 (Figures 11G,H) cells, which was supported by the wound healing assays. Collectively, these findings indicated that *MAOA i*nhibited the proliferation and migration of BLCA cells, providing a mechanistic basis for the observed association between high *MAOA* expression and improved patient prognosis.

## Discussion

Molecular understanding of BLCA biology is largely behind that of other solid cancers, which is disadvantageous for clinical care. Along with the aging population, the number of patients with BLCA is expected to increase, leading to a considerable burden on public health and healthcare systems (Van Hoogstraten et al., 2023). With the development of DNA-based genome-wide and RNA-based profiling studies, more complex tumor subtypes and disease pathogenesis are being defined, followed by the clinical requirement for precise and credible biomarkers to develop therapy strategies (Knowles and HURST, 2025). Although the NMIBC and MIBC have been well defined for many years, the biological features to predict prognosis and guide clinical therapy have not yet translated into clinical practice.

Here, we constructed a novel four-gene signature (*PATZ1*, *TTC6*, *AEBP1*, and *MAOA*) based on FAM-related genes to predict BLCA prognosis. FAM plays a central role in cancer cell proliferation and cancer progression. Based on FAM-related genes, we calculated the BLCA RNA-seq data from TCGA and obtained two clusters, followed by further identification of the prognosis-associated genes and construction of the FAMR model to predict BLCA prognosis. We then validated the FAMR model using the external validation cohort and analyzed the correlation between FAMR and the biological functions as well as clinical features.

POZ/BTB and AT hook-containing zinc finger 1 (*PATZ1*), also known as ZNF278 and MAZR, is a transcription factor that typically binds DNA and functions in chromatin modeling and transcriptional regulation (Fedele et al., 2000). *PATZ1* belongs to the POZ and Kruppel-like zinc finger (POK) family, which plays key roles in cell proliferation, senescence, apoptosis, and cancer (Lee and Maeda, 2025; Lunardi et al., 2025; Kelly and Daniel, 2025). Several studies indicate the carcinogenic role of *PATZ1*, which is consistent with our findings that high expression of *PATZ1* is correlated with poor prognosis. *PATZ1* is highly expressed in several cancers, including colon, testicular, and breast tumors (Tian et al., 2008; Fedele et al., 2008; Yang et al., 2010). *PATZ1* knockdown blocks the proliferation of colorectal carcinoma cells (Tian et al., 2008) and makes glioma cells more sensitive to apoptosis (Tritz et al., 2008). On the other hand, PATA1 knockout mice spontaneously develop tumor, including BCL6-expressing lymphomas, sarcomas, and hepatocellular carcinomas (Pero et al., 2012), with the evaluated expression of cell cycle activation-related proteins such as CDK4, HMGA1, and cyclin D2 (Valentino et al., 2013a). Mechanically, PATZA either interacts with p53 and enhances its transcription activity or binds p53-targeted genes in p53-null Saos-2 cells, which regulates transcription oppositely and results in proapoptotic and antiapoptotic activities (Valentino et al., 2013b).

Tetratricopeptide repeat domain 6 (*TTC6*) belongs to the TTC family, which is mainly involved in the formation and operation of the cilia and flagellar structures (Goebl and Yanagida, 1991; Hoffmann et al., 2022; Lorès et al., 2019). The deletion of *TTC6* causes diminished sperm motility and circular sperm swimming, leading to male subfertility in mice (Wang et al., 2091). The role of *TTC6* in cancers is rarely reported. Here, we identified *TTC6* as a prognosis-related gene in BLCA. Patients with high expression of *TTC6* had poor prognosis. However, the mechanism of *TTC6* in regulating BLCA requires further investigation.

Adipocyte enhancer-binding protein 1 (*AEBP1*) is initially identified as a transcriptional repressor and is involved in biological processes, including adipogenesis, inflammation, mammary gland development, and tumorigenesis (Majdalawieh et al., 2020). The overexpression of *AEBP1* is associated with mammary epithelial cell hyperplasia and increased proliferation of primary glioblastomas (Holloway et al., 2012; Reddy et al., 2008). Furthermore, *AEBP1* is reported to interact with IκBα in the macrophage, which is the key inhibitor of the canonical NF-κB pathway (Majdalawieh and Ro, 2010). The overexpression of *AEBP1* causes increased activation of NF-κB (Majdalawieh et al., 2007). Given the critical role of the canonical NF-κB pathway in carcinogenesis, the pro-tumorigenic function of *AEBP1* may be attributed to the NF-κB pathway. Notably, weighted gene co-expression network analysis (WGCNA)-based studies revealed a correlation between *AEBP1* and BLCA tumor progression, highlighting that high expression of *AEBP1* is correlated with better overall survival of BLCA (Li et al., 2017), which is consistent with our findings.

Monoamine oxidase A (*MAOA*) belongs to the MAO family that catalyzes the oxidative deamination of monoamine neurotransmitters and dietary amines (Han et al., 2025). *MAOA* is well known to function in the brain, and the inhibitor of *MAOA* (MAOIs) is applied for clinical neurological disorder therapy (Han et al., 2025). As for cancers, *MAOA* is reported to promote prostate cancer progression and suppress the growth of gastric cancer (Wan et al., 2025; Yin et al., 2025). Furthermore, *MAOA* is also expressed in intra-tumoral T cells and tumor-associated macrophages (TAM). Thus, targeting *MAOA* could be a multifunctional approach for tumor therapy. Indeed, *MAOA*-deficient mice have reduced B16-melanoma tumor growth and altered TAM polarization (Wang et al., 2025). The treatment of *MAOI* induces TAM reprogramming and thereby enhances the antitumor T-cell responses and suppresses tumor growth in preclinical B16-induced mouse syngeneic and human A375-induced xenograft melanoma tumor models (Wang et al., 2025). Here, we report that the expression of *MAOA* was negatively correlated with BLCA prognosis. Functional studies revealed that *MAOA* knockdown significantly promoted the proliferation and migration capacities of T24 and 5637 BLCA cells *in vitro*. These findings suggest that *MAOA* may function as a tumor suppressor in BLCA and could serve as a potential therapeutic target for BLCA treatment.

Based on the promising clinical and biological correlations demonstrated, the FAMR model developed in this study provides a novel, practical tool for prognostic stratification in BLCA. By integrating the expression patterns of four key FAM-related genes, the FAMR score effectively distinguishes high-risk patients who may benefit from more aggressive or targeted therapeutic strategies. Its validation across independent cohorts underscores its robustness and the potential for clinical translation, offering a complementary approach to existing pathological and molecular classifications.

Despite its predictive strength, in this study, we acknowledge several limitations that warrant future investigation. Foremost, although we have established the individual prognostic relevance of *PATZ1*, *TTC6*, *AEBP1*, and *MAOA*, the precise molecular mechanisms through which these four genes collectively and interactively dysregulate fatty acid metabolism to drive BLCA progression remain to be fully elucidated. In particular, the functional role of *TTC6* in cancer biology is largely unexplored, and the context-dependent tumor-suppressive or oncogenic functions of *PATZ1* and *MAOA* require deeper mechanistic validation in BLCA-specific settings. Furthermore, the current model is based on retrospective bioinformatic analysis; prospective studies on larger and more diverse patient populations are essential to confirm its clinical utility. Finally, future work integrating multi-omics data and functional experiments is crucial to decipher the causal regulatory network and to explore the therapeutic potential of targeting these genes or the FAM pathway in BLCA.

## Data Availability

The original contributions presented in the study are included in the article/Supplementary Material; further inquiries can be directed to the corresponding authors.
